# “Internet+ Recycling” Platform Participation Selection Strategy in a Two-Echelon Remanufacturing Closed-Loop Supply Chain

**DOI:** 10.3390/ijerph20053999

**Published:** 2023-02-23

**Authors:** Zhangwei Feng, Deyan Yang, Xintian Wang

**Affiliations:** 1 School of Business, Ningbo University, Ningbo 315211, China; 2 Business School, Jiangsu Normal University, Xuzhou 221116, China; 3 National Academy of Economic Strategy, Chinese Academy of Social Sciences, Beijing 100732, China

**Keywords:** “Internet+ recycling”, platform participation strategy, two-echelon remanufacturing, closed-loop supply chain management, game theory

## Abstract

Compared with traditional offline recycling channel, recycling through the “Internet+ recycling” platform has increasingly attracted the academic and practical intention in the past decade because of its accessibility and convenience. To promote the recycling initiatives and construct sustainable operations, how to stimulate supply chain stakeholders participating in the online recycling becomes a challenge issue. This paper considers one supplier, one manufacturer, and one third-party recycler (3PR) in a two-echelon remanufacturing closed-loop supply chain with an “Internet+ recycling” platform, in which consumers can access the online recycling platform and make an appointment for recycling without a physical visit. The manufacturer has three choices: either do not participate or participate with one of two strategies: *cost-sharing* (*CS*) or *active promotion* (*AP*) strategy. We develop a Stackelberg game model to study the motivation of the manufacturer to participate in the “Internet+ recycling” platform and the influence mechanism of key factors. The key findings include the following: (1) compared with the case without the “Internet+ recycling” platform, when the proportion of cost sharing for the 3PR is low, strategy *CS* contributes to the improvement of the 3PR’s performance; (2) in the presence of two participation strategies, when the disassembly rate is low enough, the manufacturer prefers strategy *AP*; otherwise, he selects strategy *CS*; and (3) a high proportion of cost sharing for the manufacturer or low promotion effort cost can increase the whole profit of the closed-loop supply chain.

## 1. Introduction

The wastage in electronic products, such as used or second-hand computers and mobile phones, has drawn significant attention worldwide because of its influence on sustainable operations [1,2]. According to the **Dutch Waste Electrical and Electronic Equipment** (**Dutch WEEE**) Flows report in 2020, electrical and electronic equipment wastage has been increased from 324 kt in 2010 to 366 kt in 2018, among which around half of this waste can be recycled. More seriously, about 16 million electronic products have been discarded worldwide in 2020, including mobile phones, printers, air conditioners, TVs, refrigerators, etc. which generated about 800 million tons of solid waste, and this figure is even underestimated [3]. Dutch WEEE indicates that approximately one-quarter of the wastage which was disposed or exported has not been documented or calculated in the report [4]. To deal with this huge wastage and avoid environmental pollution, an efficient recycling is therefore emerged as an urgent and challenging issue [5,6]. Governments have made significant efforts on promoting the recycling initiatives; for example, China has committed to “carbon neutrality” and promulgated the Action Plan for Carbon Dioxide Peaking before 2030 and Carbon Neutral before 2060 in 2021 [7]. This plan proposes to promote the low-carbon transformation of vehicles and equipment, and it stimulates the adoption of clean energy and green technologies including efficient and convenient recycling operations [8].

### 1.1. Research Background

Digital economy and internet technologies (5G, AI, Blockchain, etc.) have received unprecedented development and become one of the significant driven factors to push the transformation of global market economy and business operations all over the world [9]. In the **closed-loop supply chain** (**CLSC**) management and remanufacturing operations research field, multi-channel recycling including “Internet+” recycling platforms and traditional recycling channels have emerged as an important strategy to promote the recycling system of renewable resources [10,11]. For example, “Aihuishou”, the biggest O2O (Online to Offline) electronic products collecting internet platform in China, has been in operation for ten years with over 30,000,000 customers.

Practically, manufacturers may be willing to collect disposed electronic products to produce remanufactured products in CLSCs because of the economic benefits and environmental activism [12]. However, given the consideration that a proportion of recycled electronic products with dysfunctional parts cannot be used for remanufacturing products [13] and recycling business is usually not the core business of manufacturers [14], outsourcing the recycling business to a professional **third-party recycler** (**3PR**), especially the one with Internet+ technologies, is considered as an efficient strategy aiming to improve the competitiveness of enterprises’ core business and save costs [15].

### 1.2. Research Motivation

While many studies have discussed two-echelon remanufacturing processes in CLSCs [13,16], few have discussed both modes by discussing which remanufacturing strategy is suitable for manufacturers and suppliers [15]. Moreover, the studies investigating both the economic and environmental characteristics of the 3PR in the “Internet+ recycling” platform remain scant. In our study, we analyze the scenario as follows.

(1) The 3PR recycles the disposed electronic products from consumers, disassembles, and inspects the quality of the parts;
(2) Then, the 3PR resells the high-quality parts to manufacturers to produce new products and resells the low-quality parts to suppliers as raw materials to produce new parts [17].

Given the scenario above, the costs of the 3PR therefore are composed of recycling cost, disassembling cost, and recovery efforts costs.

To motivate the recycling effort of the 3PR, the manufacturer can choose to undertake a certain percentage of the recycling cost of the 3PR, namely the **cost-sharing** (***CS***) strategy. Additionally, some manufacturers can also choose to participate in the “Internet+ recycling” platform operated by the 3PR by paying to the 3PR based on the 3PR recycling promotion efforts, namely the **active promotion** (***AP***) strategy. We assume that under the strategy *AP*, the “Internet+ recycling” platform jointly built by the manufacturer and the 3PR can not only promote the recycling of waste electronic products but also increase the sales of new products because of its reputation on environmental protection.

In our study, we discuss three scenarios, including the no-participation scenario, cost-sharing contract, and active promotion policy. We aim to identify the economic driving forces of the manufacturers to participate in the “Internet+ recycling” platform and promote the recycling business.

### 1.3. Research Questions

Based on the above research background and motivation, we provide the research questions of this paper as follows.

RQ1: What are the key differences for manufacturers to participate in the “Internet+ recycling” platform with different participation strategies (strategies *CS* and *AP*)?

RQ2: How do the key factors (i.e., disassembly rate, cost-sharing proportion of recycling cost, etc.) affect the participation strategies decisions of manufacturers?

### 1.4. Research Structure

The remainder of the paper is organized as follows. Section 2 reviews the related literature. Section 3 introduces the method and model setup. Section 4 provides an equilibrium analysis of basic model, and Section 5 compares the outcomes of the extension model and the basic model. Section 6 discusses managerial insights and projects the future directions.

## 2. Literature Review

Two streams of research are closely related to our study: (1) two-echelon remanufacturing CLSC; and the (2) “Internet+ recycling” platform and cost-sharing contract.

### 2.1. Two-Echelon Remanufacturing CLSC

Many suppliers and manufacturers authorize 3PRs to take lead of the recycling operations of disposed electronic products and try to motivate the 3PR to disassemble the collected products either for products or parts remanufacturing, which is of great importance in reducing environment pollution and promoting the reuse of resources [18]. However, in the remanufacturing CLSC context, some scholars believe that only the supplier–remanufacturing relationship is important and needs to be analyzed [19,20], whereas other studies argue that two-echelon remanufacturing including the supplier-remanufacturing and manufacturer-remanufacturing relationships are valuable to be comprehensively discussed and compared [16,21,22]. However, despite its significance, the two-echelon remanufacturing CLSC has not yet been investigated thoroughly in the extant literature. We only identify a few studies focusing on the remanufacturing issue in the context of two-echelon remanufacturing CLSC. For example, Feng et al. [13] integrate the buyback price, effective recovery ratio, and recycling effort level in a CLSC with two-echelon remanufacturing. Xiang and Xu [23] propose a two-stage remanufacturing CLSC dynamic model consisting of a manufacturer, an **Internet Recycling Platform** (**IRP**), and a supplier. This work identifies that the IRP’s overconfident behavior is beneficial to itself but damages the supplier’s profit. Notably, although the IRP’s overconfidence and cost-sharing strategies may decrease the supplier’s profit, the total profit of the CLSC increases. Zhang et al. [24] investigate which party should take lead of the raw materials collection in a two-echelon remanufacturing and discuss the environmental benefit, economic benefit, and social welfare accordingly.

Distinct from the above research, our study introduces a third-party “Internet+ recycling” platform in the CLSCs and discusses the trade-offs of different strategies. We focus on how the adoption of the “Internet+ recycling” platform and its investment cost affect the remanufacturing operations of the manufacturer.

### 2.2. “Internet+ Recycling” Platform and Cost-Sharing Contract

The “Internet+” recycling program has been developed rapidly in China recently [25,26]. Researchers pay increasingly attention on related topics. For instance, Jian et al. [26] study collaborative collection strategies in a recycling system composed of a third-party recycler and an e-tailer recycler in the “Internet+ recycling” business model. This work discusses the collaboration and mutual influences of partners on the recycling disposed products. In the context of CLSC, researchers focus on the cost-sharing mechanism selection under different channel power structures [27,28,29]. Particularly, Chen et al. [29] establish a CLSC integrating remanufacturing process innovation and the cost-sharing mechanism and examine remanufacturing process innovation, pricing decisions, and the cost-sharing mechanism under different power structures of the CLSC. 

Although the cost-sharing mechanism in CLSCs has been widely analyzed in the existing literature [27,29,30], few studies investigate the trade-offs between the cost-sharing mechanism and actively promotion strategy in the context of the “Internet+ recycling” platform in CLSCs. To fill this gap, we employ game models to investigate how the introduction of a cost-sharing mechanism or active promotion strategy for the “Internet+ recycling” platform affect the profits. A more detailed classification and differences of the literature are presented in Table 1, which includes four features: research object, research method, recycling channel or platform, and main features. The last row of Table 1 provides the main features of this study.

### 2.3. Theoretical Contribution

After scrutinizing the extant literature, we identify that studies focusing on the influence of driven factors on the recycling in a two-echo remanufacturing CLSC remain scant. One exception is the study of Feng et al. [13]; this work investigates the recycling decisions of the 3PR in a CLSC with a two-echelon remanufacturing scenario and integrates price, effective recycling ratio, and recycling effort level within a modeling framework. Their study, however, does not consider the recovery factors that influence the uptake of introducing the “Internet+ recycling” platform, which is a key focus of our paper. Owing to the differences in the framework design (transferring a 3PR to an “Internet+ recycling” platform in our study), this paper fills a research gap by investigating when a manufacturer should select a cost-sharing strategy over the active promotion strategy given the consideration of both economic benefits and environmental responsibility.

The novel areas of this research of the work are as follows.

(1) We design an “Internet+ recycling” platform in a two-echelon remanufacturing CLSC with two participation strategies and study the interaction between recovery factors (disassembly rate, cost-sharing proportion of recycling cost, etc.) and “Internet+ recycling” platform participation strategies in the CLSC.
(2) In the model settings, we discuss the effects of the disassembly rate and different buyback fees and describe the consumption demand and recycling quantity stimulated by the “Internet+ recycling” platform.

According to our assumptions and analysis, the key findings and innovative conclusions are drawn below.

(1) In the presence of the “Internet+ recycling” platform, a low disassembly rate plays an inhibitory role on the profits of suppliers, whereas it increases the profits of other firms.
(2) A lower cost-sharing proportion of recycling cost for the 3PR contributes the profit 3PR, and a higher cost-sharing proportion of recycling cost for the 3PR or a lower “Internet+ recycling” cost undertaken by the manufacturer can increase the total profit of the CLSC.

## 3. Method and Model Setup

### 3.1. System Description and Assumptions

**Figure 1:**
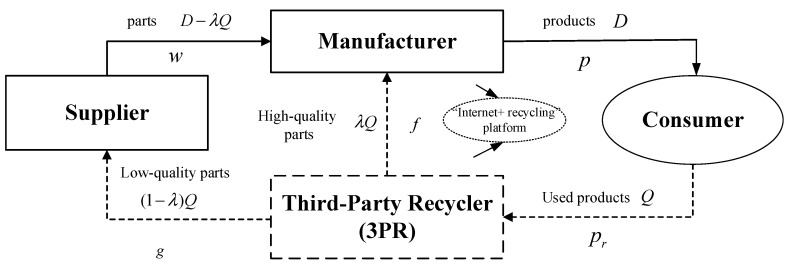
A two-echelon remanufacturing CLSC with an “Internet+ recycling” platform.

This paper develops a closed-loop supply chain (CLSC) with one supplier (S), one manufacturer (M), and one third-party remanufacturer (3PR). In forward logistics, the supplier produces new parts and wholesales them to the manufacturer, and then, the manufacturer produces new products and sells them to consumers. In reverse logistics, the 3PR recycles used products from consumers, disassembling the used products into high-quality (low-quality) parts and reselling them to the manufacturer (the supplier). The manufacturer is the Stackelberg leader and chooses whether to invest the cost of popularizing the “Internet+ recycling” platform. Figure 1 shows an “Internet+ recycling” platform, which is popularizing by both the 3PR and the manufacturer. Table 2 provides a summary of all parameters and variables used in the research.

**Assumption 1.**  *It is assumed that remanufactured parts are homogeneous with new parts, and analogously, remanufactured products are homogeneous with the new products. In addition, there is a sufficient quantity of wasted new products for recycling or manufacturing [33,34].*

**Assumption 2.**  *End-of-life or used products can be disassembled into high-quality parts and low-quality parts, and the former can be produced as new parts while the latter can be produced as raw material [13,23].*

**Assumption 3.**  *We denote λ as the disassembly rate for used products, and it is exogenous. The 3PR should take p_r_ + dλ of the per unit used product for recycling from consumers and disassembling them into high-quality parts [13].*

**Assumption 4.**  *Similar to Zhang et al. [24], we assume w > f > p_r_ > g to ensure economic motivation for firms in the recycling process and in line with the real situation of recycling.*

### 3.2. Demand and Recycling Quantities

In the basic model (cost-sharing contract, *CS*), the demand function is D(p)=α−βp [13,24]. Recycling quantities of used products are $Q(p_{r},A_{t})=ap_{r}+b_{1}A_{t}$ with the “Internet+ recycling” cost $C_{A_{t}}=\theta_{2}A_{t}^{2}/2$ for the 3PR [26].

### 3.3. Firm Problem and Sequence

Given the above assumptions, the supplier’s profit under scenario *CS* is

(1) $$\pi_{S}^{CS}=(w-m-c_{s})(D-\lambda Q)+(m-g)(1-\lambda )Q$$

where *w* – *m* − *c_s_* is the marginal profit of per unit new parts and *m* − *g* is the marginal cost savings from low-quality parts.

The manufacturer’s profit under scenario *CS* is

(2) $$\pi_{M}^{CS}=(p-w-c_{m})D+(w-f)\lambda Q-(1-\delta )C_{A_{t}}$$

where *p* – *w* − *c_m_* is the marginal profit of per unit new products, *w* − *f* is the marginal cost savings from high-quality parts, and $(1-\delta )C_{A_{t}}$ is the recycling cost shared by the manufacturer.

The 3PR’s profit under scenario *CS* is

(3) $$\pi_{3PR}^{CS}=f\lambda Q+g(1-\lambda )Q-\delta C_{A_{t}}$$

where $\delta C_{A_{t}}$ is the recycling cost shared by the 3PR.

The sequence of decisions in this game is

*(i)*  *The supplier announces the wholesale price w;*
*(ii)*  *The manufacturer decides the sale price p;*
*(iii)*  *Observing above, the 3PR determines the recycling price p_r_ and recycling effort level A_t_.*

**Figure 2:**
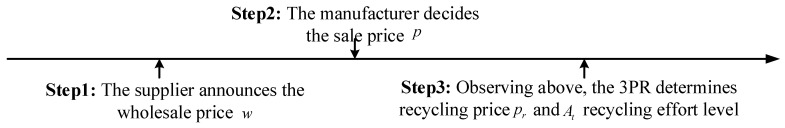
The flow diagram of this game sequence in the CLSC with cost-sharing contract.

Figure 2 represents the flow diagram of this game sequence in the CLSC with a cost-sharing contract. Using backwards induction, firms can obtain the equilibrium decisions. 

## 4. Basic Model and Analysis—Scenario of Cost-Sharing Contract (*CS*)

### 4.1. The Equilibrium Decisions under Scenario CS

**Lemma 1.**  *Assume *$b_{1}<\sqrt{a\theta_{1}}$*. We have *$w_{}^{CS*}=[\alpha +\beta (c_{s}+m-c_{m})-2a^{2}\theta_{1}\lambda N]/(2\beta )$*, *$p_{}^{CS*}=[\alpha +(c_{m}+w_{}^{CS*})\beta ]/(2\beta )$*, *$p_{r}^{CS*}=(a\theta_{1}-b_{1}^{2})N$*, and *$A_{t}^{CS*}=ab_{1}N$*, where *N=[g(1−λ)+(f−dδ)λ]/(δX)>0*and *$X=2a\theta_{1}-b_{1}^{2}>0$*. Furthermore, we obtain *$Q_{}^{CS*}$*, *$D_{}^{CS*}$*, *$\pi_{3PR}^{CS*}$*, *$\pi_{S}^{CS*}$*, and *$\pi_{M}^{CS*}$.

Proof of lemmas and corollaries are provided in Appendix A, Appendix B, Appendix C, Appendix D, Appendix E and Appendix F.

Note that the assumption of $b_{1}<\sqrt{a\theta_{1}}$ ensures the existence and uniqueness of the optimal decisions. Particularly, when the manufacturer does not participate in the “Internet+ recycling” platform, we have Lemma 2.

**Lemma 2.**  *Assume *$b_{1}<\sqrt{a\theta_{1}}$*. We have *$w_{}^{N*}=[\alpha +\beta (c_{s}+m-c_{m})-2a^{2}\theta_{1}\lambda M]/(2\beta )$*, *$p_{}^{N*}=[\alpha +(c_{m}+w_{}^{N*})\beta ]/(2\beta )$*, *$p_{r}^{N*}=(a\theta_{1}-b_{1}^{2})M$*, and *$A_{t}^{N*}=ab_{1}M$*, where *M=[g(1−λ)+(f−d)λ]/X>0*, *$X=2a\theta_{1}-b_{1}^{2}>0$*, and *M<N*. Furthermore, we obtain *$D_{}^{N*}$*, *$Q_{}^{N*}$*, *$\pi_{S}^{N*}$*, *$\pi_{M}^{N*}$*, and *$\pi_{3PR}^{N*}$.

According to the sensitivity analysis of the disassembly rate to the 3PR’s equilibrium decisions, when $f>{\hat{f}}_{1}=g+d$, we have $\partial Q_{}^{N*}/\partial \lambda >0$ and $\partial \pi_{3PR}^{N*}/\partial \lambda >0$; otherwise, $\partial Q_{}^{N*}/\partial \lambda \leq 0$ and $\partial \pi_{3PR}^{N*}/\partial \lambda \leq 0$. It implies that whether the high disassembly rate is beneficial to the recycling quantities and the profit of the 3PR depends on unit buyback fees for high-quality (low-quality) parts and unit processing cost. Particularly, only when the unit buyback fee for high-quality parts is high enough (i.e., $f>{\hat{f}}_{1}$ ) can stimulate the 3PR to recycle and disassemble used products, which is in line with reality. Otherwise, the 3PR has no economic drive to do so.

In addition, with the increase in the sensitivity coefficient of recycling price and recycling effort level, the demand is also increased (i.e., $\partial D_{}^{N*}/\partial a>0$ and $\partial D_{}^{N*}/\partial b_{1}>0$ ), which is not an intuitive result. It further suggests that when recycling used products by the “Internet+ recycling” platform built by the 3PR independently, consumers perceive the environmental performance of the used products and show their own social responsibility, which also promotes their purchase of new products from manufacturers. However, this promotion effect might be very limited. It is possible that if the manufacturer participates in the “Internet+ recycling” platform through strategy *CS* or *AP*, this positive promotion effect could be more obvious. 

In fact, Lemma 2 is a special case of Lemma 1 (i.e., *δ* = 1); that is, when the recycling cost is fully borne by the 3PR, the manufacturer does not participate in the “Internet+ recycling” platform. We find that only when the unit buyback fee for high-quality parts is high enough (i.e., $f>{\hat{f}}_{2}=g+d\delta$) can it stimulate the 3PR to recycle and disassemble used products (i.e., when $f>{\hat{f}}_{2}$, $\partial Q_{}^{CS*}/\partial \lambda >0$ and $\partial \pi_{3PR}^{CS*}/\partial \lambda >0$ ), which is robust to the result of Lemma 1. However, at this time, the motivation (i.e., threshold value) of the 3PR for recycling and disassembling is higher than that of the no-participation scenario (i.e., ${\hat{f}}_{2}<{\hat{f}}_{1}$ ), as there is no need to pay a higher recycling price for high-quality parts.

### 4.2. The Analysis under Scenario CS

Lemmas 1 and 2 derive Corollaries 1 and 2.

**Corollary 1.**  $w_{}^{CS*}<w_{}^{N*}$*,*$p_{}^{CS*}<p_{}^{N*}$*,*$A_{t}^{CS*}>A_{t}^{N*}$*,*$p_{r}^{CS*}>p_{r}^{N*}$*,*$D_{}^{CS*}>D_{}^{N*}$*,*$Q_{}^{CS*}>Q_{}^{N*}$.

Corollary 1 implies that strategy *CS* promotes the reduction of wholesale and sale prices of new products, and it further increases the demand of new products (i.e., $w_{}^{CS*}<w_{}^{N*}$, $p_{}^{CS*}<p_{}^{N*}$, and $D_{}^{CS*}>D_{}^{N*}$ ). It also increases the recycling price of used products, the recycling effort level, and the recycling quantities (i.e., $p_{r}^{CS*}>p_{r}^{N*}$, $A_{t}^{CS*}>A_{t}^{N*}$, and $Q_{}^{CS*}>Q_{}^{N*}$ ). This reveals that the manufacturer adopting strategy *CS* to participate in the “Internet+ recycling” platform can not only promote their own new product sales but also promote the recycling quantities of used products, which plays a multi-win situation in occupying market quantity share, promoting products, and promoting circular economy. However, it does not mean that the 3PR or the manufacturer benefits from strategy *CS*, which needs further comparisons.

**Corollary 2.**  *When* $\delta <\min\{1,{\hat{\delta }}_{2}\}$ *, the 3PR prefers strategy CS (i.e.,* $\pi_{3PR}^{CS*}>\pi_{3PR}^{N*}$ *); otherwise, it prefers the manufacturer to stay out of the “Internet+ recycling” platform.*

When the cost-sharing proportion of recycling cost for the 3PR is relatively small (i.e., $\delta <\min\{1,{\hat{\delta }}_{2}\}$ ), the 3PR is motivated to prefer the strategy *CS* (i.e., $\pi_{3PR}^{CS*}>\pi_{3PR}^{N*}$ ). Otherwise, the 3PR would rather the manufacturer not participate in the “Internet+ recycling” platform. Because when the manufacturer shares in part of the 3PR’s recycling costs, it results in reducing the 3PR’s operating costs, and it further improves the opportunity for the 3PR to obtain a higher profit. At this point, the manufacturer taking the strategy *CS* to participate in the “Internet+ recycling” platform is superior to not participating in the “Internet+ recycling” platform, which is a win–win situation.

## 5. Extended Model and Analysis—Scenario of Actively Promotion Policy (*AP*)

In the extended model (**actively promotion policy, *AP***), the recycling quantities of used products are $Q(p_{r},A_{t},A_{m})=ap_{r}+b_{1}A_{t}+b_{2}A_{m}$ [15], where *A_m_* is the recycling effort level by the manufacturer with the recycling cost $C_{A_{m}}=\theta_{1}A_{m}^{2}/2$. Consumers can realize the manufacturer’s social responsibility and return used products when the manufacturer participating “Internet+ recycling” platform [26].

Given the above assumptions, the supplier’s profit under scenario *AP* is

(4) $$\pi_{S}^{AP}=(w-m-c_{s})(D-\lambda Q)+(m-g)(1-\lambda )Q$$

where *w* – *m* − *c_s_* is the marginal profit of per unit new parts and *m* − *g* is the marginal cost savings from low-quality parts.

The manufacturer’s profit under scenario *AP* is

(5) $$\pi_{M}^{AP}=(p-w-c_{m})D+(w-f)\lambda Q-C_{A_{m}}$$

where *p* – *w* – *c_m_* is the marginal profit of per unit new products, *w* − *f* is the marginal cost savings from high-quality parts, and $C_{A_{m}}$ is the “Internet+ recycling” cost undertaken by the manufacturer.

The 3PR’s profit under scenario *AP* is

(6) $$\pi_{3PR}^{AP}=f\lambda Q+g(1-\lambda )Q-C_{A_{t}}$$

where $C_{A_{t}}$ is the recycling cost undertaken by the 3PR.

The sequence of decisions in the game is

*(i)*  *The supplier announces the wholesale price w;*
*(ii)*  *The manufacturer decides the sale price p and recycling effort level A_m_;*
*(iii)*  *Observing the above, the 3PR determines recycling price p_r_ and recycling effort level A_t_.*

**Figure 3:**
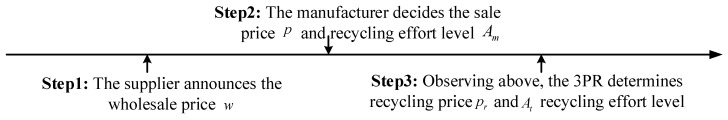
The flow diagram of this game sequence in the CLSC with an active promotion policy.

Figure 3 represents the flow diagram of this game sequence in the CLSC with the active promotion policy. Using backwards induction, firms can obtain the equilibrium decisions.

**Lemma 3.**  *Assume *$b_{1}<\sqrt{a\theta_{1}}$*and *$\gamma <\min\{\sqrt{2\beta \theta_{2}},(\beta \theta_{2}X^{2}+2Z^{2})/(2XZ)\}$*. We have *$w_{}^{AP*}=[\alpha +\beta (m+c_{s}-c_{m})]\cdot (2XZ\gamma -2Z^{2}-\beta \theta_{2}X^{2})+a\theta_{1}\{b_{2}(\gamma X-2Z)[(\alpha -c_{m}\beta -f\beta )\lambda -(m-g)\beta (1-\lambda )]-aX^{2}YM\}$*, *$p_{}^{AP*}=\{[\alpha \theta_{2}+(\beta \theta_{2}-\gamma^{2})(w_{}^{AP*}-c_{m})]X+\gamma Z(w_{}^{AP*}-f)\}/(XY)$*, *$A_{m}^{AP*}=\{\gamma [\alpha +\beta (w_{}^{AP*}-c_{m})]X+2\beta Z(w_{}^{AP*}-f)\}/(XY)$*, *$p_{r}^{AP*}=(a\theta_{1}-b_{1}^{2})M-\theta_{1}b_{2}A_{m}^{AP*}/X$*, and *$A_{t}^{AP*}=ab_{1}M+b_{1}b_{2}A_{m}^{AP*}/X$*, where *$Y=2\beta \theta_{2}-\gamma^{2}>0$* and *$Z=ab_{2}\theta_{1}\lambda >0$*. Furthermore, *$D_{}^{AP*}$*, *$Q_{}^{AP*}$*, *$\pi_{S}^{AP*}$*, *$\pi_{M}^{AP*}$*, and *$\pi_{3PR}^{AP*}$.

Note that the assumptions of $b_{1}<\sqrt{a\theta_{1}}$ and $\gamma <\min\{\sqrt{2\beta \theta_{2}},(\beta \theta_{2}X^{2}+2Z^{2})/(2XZ)\}$ ensure the existence and uniqueness of the optimal decisions. In addition, with a higher recycling effort level by the manufacturer, the 3PR is more profitable as $\pi_{3PR}^{AP*}=\theta_{1}{\left\{b_{2}A_{m}^{AP*}+a[g+(f-d-g)\lambda ]\right\}}^{2}/(2X)$. From Lemmas 1 and 3, we obtain the motivation and preference for the manufacturer participating in the “Internet+ recycling” platform.

Lemmas 1 and 3 derive Corollary 3.

**Corollary 3.**  *There is a threshold* ${\hat{\delta }}_{3}$ *that when* $\delta >{\hat{\delta }}_{3}$ *, the strategy AP is better than the strategy CS (i.e.,* $\pi_{3PR}^{AP*}>\pi_{3PR}^{CS*}$ *).*

Compared to the no-participation scenario, if the manufacturer participates in the “Internet+ recycling” platform with strategy *AP*, the 3PR is also beneficial (i.e., $\pi_{3PR}^{AP*}>\pi_{3PR}^{CS*}$ ); i.e., the participation of the manufacturer indirectly motivates the 3PR to participate in the recycling and remanufacturing process. However, compared to strategy *CS*, whether the 3PR prefers strategy *CS* mainly depends on the cost-sharing proportion. When it is high (i.e., $\delta >{\hat{\delta }}_{3}$ ), the 3PR’s profit is compressed significantly, and the 3PR is preferred to strategy *AP*.

**Figure 4:**
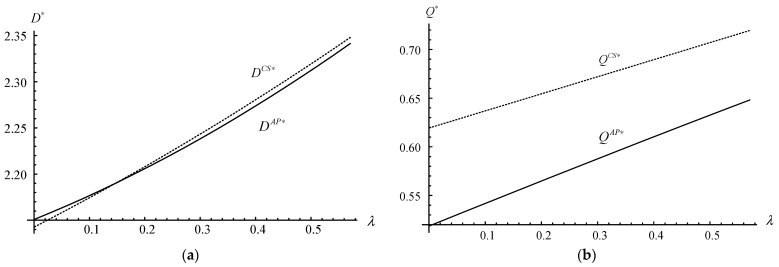
Demand/Recycling quantities vs. the disassembly rate. (**a**) Demand vs. the disassembly rate. (**b**) Recycling quantities vs. the disassembly rate.

**Figure 5:**
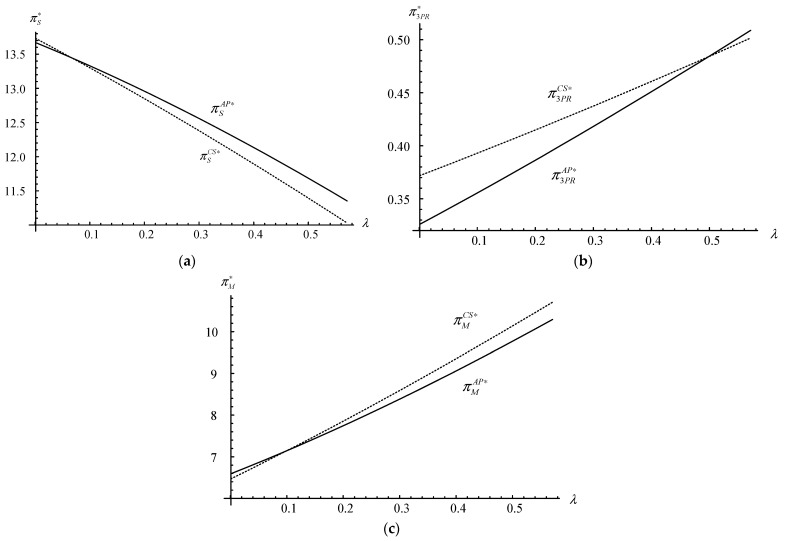
The firms’ profits vs. the disassembly rate. (**a**) The supplier’s profit vs. the disassembly rate. (**b**) The 3PR’s profit vs. the disassembly rate. (**c**) The manufacturer’s profit vs. the disassembly rate.

**Figure 6:**
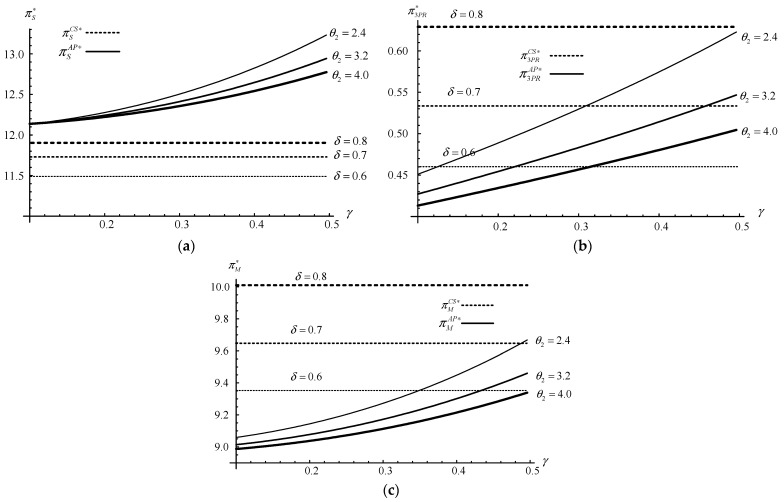
The firms’ profits vs. *θ*_2_, *δ*, *γ*. (**a**) The supplier’s profit vs. *θ*_2_, *δ*, *γ*. (**b**) The 3PR’s profit vs. *θ*_2_, *δ*, *γ*. (**c**) The manufacturer’s profit vs. *θ*_2_, *δ*, *γ*.

As the equilibrium solutions under strategy *AP* are too complex, many decisions and profits of manufacturers cannot be directly compared with the results of those under the no-participation scenario or strategy *CS*. Furthermore, some innovative management hints are needed from numerical studies. Therefore, we conduct several numerical studies (based on Mathematica 8.1) to supplement the previous research conclusions. The default values are as follows: *c_m_* = 1.5, *c_s_* = 1.2, *m* = 2.2, *α* = 12, *f* = 1.7, *δ* = 0.8, *λ* = 0.4, *β* = 0.7, *d* = 0.2, *g* = 1.2, *γ* = 0.1, *θ*_1_ = 3.2, *θ*_2_ = 2.4, *a* = 0.8, *b*_1_ = 0.4, and *b*_2_ = 0.35. These default values are selected as they firstly satisfy the modelling assumptions (i.e., $b_{1}<\sqrt{a\theta_{1}}$ and $\gamma <\min\{\sqrt{2\beta \theta_{2}},(\beta \theta_{2}X^{2}+2Z^{2})/(2XZ)\}$ ) and then allow us to explore the trade-offs and managerial insights in more detail. Furthermore, the numerical analysis has been implemented multiple times with different parameters to increase its robustness and credibility [35]. Figure 4, Figure 5 and Figure 6 illustrate the impacts of *λ*, *θ*_2_, *δ*, and *γ* on the demand, recycling quantities, and firms’ profit under strategies *N*, *CS*, and *AP*.

Figure 4a,b reveal that the demand of new products and the recycling quantities of used products are increasing with the increase in the disassembly rate (i.e., $\partial D_{}^{j*}/\partial \lambda >0$ and $\partial Q_{}^{j*}/\partial \lambda >0$, where j=CS,AP ). Because with the increase in the disassembly rate, both the proportion and quantities of high-quality parts increase. It improves the profit of dismantling and motivates the 3PR to recycle and disassemble more quantities of used products. Furthermore, the increase in recycling quantities of used products in the reverse logistic, to some extent, also stimulates and forces the demand of new products in the forward logistic.

In addition, the recycling quantities of used products under scenario *CS* are always higher than those under scenario *AP* (i.e., $Q_{}^{CS*}>Q_{}^{AP*}$ ). While for the demand of new products, it depends on the disassembly rate. Particularly, when the disassembly rate is very small (i.e., *λ* < 0.15), the demand of new products under scenario *AP* is higher than that under scenario *CS* (i.e., $D_{}^{AP*}>D_{}^{CS*}$ ); otherwise, $D_{}^{AP*}\leq D_{}^{CS*}$. This potentially shows that the disassembly rate under the strategy *CS* has a more significant impact on the demand of new products and the recycling quantities of used products than those under the strategy *AP* (i.e., $Q_{}^{CS*}>Q_{}^{AP*}$ and $\partial D_{}^{CS*}/\partial \lambda >\partial D_{}^{AP*}/\partial \lambda$ ).

Observing Figure 5a–c, we can obtain Results 1 and 2.

**Result 1.**  *The disassembly rate has an inhibitory effect on the supplier, while it has a promoting effect on the manufacturer and the 3PR (i.e.,*$\partial \pi_{S}^{j*}/\partial \lambda <0$*,*$\partial \pi_{M}^{j*}/\partial \lambda >0$*, and*$\partial \pi_{3PR}^{j*}/\partial \lambda >0$*, wherej* = *CS,* *AP)*.

With the increase in the disassembly rate, both the proportion and quantities of high-quality parts are increased, which motivates the 3PR to recycle and disassemble more quantities of used products; moreover, the demand of new products and recycling quantities of used products are also increased. Therefore, the 3PR obtains a higher profit by increasing buyback fees after dismantling, although the supplier and the manufacturer pay more buyback fees, the margins profits and demands of new products increased, and so did profits.

**Result 2.**  *When λ* > 0.1*, the manufacturer selects strategy CS (i.e.,* $\pi_{M}^{AP*}<\pi_{M}^{CS*}$ *); otherwise, it selects strategy AP.*

The disassembly rate has different promotion effects on the manufacturer and on the 3PR under different platform participation strategies. For the manufacturer, when the disassembly rate is not sufficiently small, the manufacturer selects strategy *CS* and increasingly prefers a higher disassembly rate (i.e., when *λ* > 0.1, $\pi_{M}^{AP*}<\pi_{M}^{CS*}$ and $\partial \pi_{M}^{AP*}/\partial \lambda <\partial \pi_{M}^{CS*}/\partial \lambda$ ). While for the 3PR, only when the disassembly rate is not very high does the 3PR prefer strategy *CS* (i.e., when *λ* > 0.5, $\pi_{3PR}^{AP*}<\pi_{3PR}^{CS*}$ ). This means that the dismantling rate is a boost for both the manufacturer and 3PR, but under the premise of a certain dismantling rate, strategy *AP* is more beneficial to the 3PR, while strategy *CS* is more beneficial to the manufacturer; they are both better off in situations where the opposite side bears more of the cost.

However, a high disassembly rate also means that the output of low-quality parts decreases, and the profit of the supplier in manufacturing parts decreases significantly. Especially when the increase in the recycling quantities of used products cannot prevent the output of low-quality parts from decreasing, or the increase in the demand of new products cannot improve the whole profit of the CLSC, the supplier can only rely on raw materials at the original price to produce new parts. Due to the inability to save more production costs or generate more profits, the supplier’s profit will obviously decrease with the increase in the dismantling rate.

Observing Figure 6a–c, we can obtain Result 3.

**Result 3.**  *The firms’ profits are increased with* δ *and* γ *but decreased with* $\theta_{2}$ *(i.e.,* $\pi_{i}^{CS*}|{}_{\delta =0.8}>\pi_{i}^{CS*}|{}_{\delta =0.7}>\pi_{i}^{CS*}|{}_{\delta =0.6}$ *,* $\partial \pi_{i}^{AP*}/\partial \theta_{2}<0$ *, and* $\partial \pi_{i}^{AP*}/\partial \gamma >0$ *, where* i=S,M,3PR *).*

The two platform participation strategies are compared by adjusting three key parameters (i.e., *θ*_2_, *γ*, and *δ*), and Result 3 shows the following: (1) a higher cost-sharing proportion of recycling costs for the 3PR can improve the profits of the supplier, the manufacturer and the 3PR as well as the whole profits of the CLSC under strategy *CS* (i.e., $\pi_{i}^{CS*}|{}_{\delta =0.8}>\pi_{i}^{CS*}|{}_{\delta =0.7}>\pi_{i}^{CS*}|{}_{\delta =0.6}$, where *i* = *S*, *M*, 3*PR*), which extends the conclusion of Corollary 2; (2) the low scaling parameter of recycling cost by the manufacturer and high sensitivity coefficient of recycling effort level by the manufacturer to demand can promote the profits of each firm (i.e., $\partial \pi_{i}^{AP*}/\partial \theta_{2}<0$ and $\partial \pi_{i}^{AP*}/\partial \gamma >0$, where *i* = *S*, *M*, 3*PR*).

Management insights: If the manufacturer expects to participate in the “Internet+ recycling” platform with the cost-sharing contract, then it needs to cooperate with the 3PR to find a lower cost-sharing proportion for the manufacturer to seek individual benefits. If the manufacturer prefers an active promotion policy, then the promotion costs should be reduced or the promotion incentive role in the demand should be increased by some effective methods.

## 6. Conclusions

### 6.1. Theoretical Implications

In our study, we consider a remanufacturing CLSC composed of a supplier, a manufacturer, and a 3PR, in which the manufacturer is the leader. We develop two game models to investigate the influence of recovery factors (disassembly rate, cost-sharing proportion of recycling cost, etc.) on the manufacturer’ decisions on whether to participate in the “Internet+ recycling” platform. We analyze three different scenarios: no participation, cost-sharing contract, and active promotion policy. We compare the investor’s decisions on introducing the “Internet+ recycling” platform under different scenarios, and the key findings of this study are as follows.

- Compared with the no-participation scenario, adopting the strategy *CS* can reduce the price of new products, increase the recycling price of used products, and promote the demands of new products and the recycling quantities of used products. Only when the cost-sharing proportion of the 3PR is low, the strategy *CS* contributes to the profit increase in the 3PR;
- In the presence of two participation strategies, the strategy *CS* is more conducive to the recycling quantities of used products;
- The effect of different strategies on the demand of new products depends on the disassembly rate. Particularly, when the disassembly rate is small (high), the demand for new products under the strategy *AP* (*CS*) is higher;
- While the low disassembly rate has a negative effect on the profit of the supplier, it increases the profits of the manufacturer and the 3PR. In addition, when the dismantling rate is low, both the supplier and 3PR prefer strategy *AP*, whereas the manufacturer prefers strategy *CS*;
- A higher cost-sharing proportion can stimulate the manufacturer to choose the strategy *CS*, whereas a lower cost of promotion effort or a higher promotion effect can motivate the manufacturer to choose the strategy *AP*. Either a higher cost-sharing proportion or a lower cost of promotion effort can contribute to the total profit of the CLSC.

### 6.2. Managerial Implications

Our findings have practical implications to manufacturers by helping manufacturers decide whether to participate in the “Internet+ recycling” platform and which participation strategy to introduce. In summary, some managerial insights and practical implications are as follows.

- The proposed “Internet+ recycling” model provides a new decision-making perspective and an optimal operational management guidance for most enterprises in the CLSCs regarding the decisions on whether to participate in recycling, especially in the Internet era;
- The conditions that help the manufacturers find the trade-offs between the active promotion policy and cost-sharing contract provide a guideline for manufacturers when deciding whether to participate in the “Internet+ recycling” platform;
- By exploring the influence mechanism of correlation coefficient on decisions and platform participation strategy, our findings can guide suppliers, manufacturers, and 3PRs to identify their optimal decisions related to platform participation strategy selection and cope with the challenges from increase in costs and different consumer preference.

### 6.3. Limitations and Future Research

Although we make efforts to make our models in line with the reality, there are still some limitations that can be investigated in future research. Firstly, we assumed that there is no difference between new and remanufactured products. However, given the different segments of consumers, such as green consumers and traditional consumers, the heterogeneous preferences for new and remanufactured products can exist and should be further studied. Secondly, incorporating the low carbon preference and carbon tax policy and different participate strategies in the CLSC might lead to new management insights. Finally, our study did not consider cap-and-trade regulation, which may be another important mechanism to be considered in the CLSC context.

## Figures and Tables

**Table 1:** Comparison of this study and related literature.

Studies	Research Object	Research Method	Recycling Channel or Platform	Main Features
Feng et al. [13]	Two-echelon re-CLSC	Game theory	Recycling by the 3PR	Recycling decisions in a two-echelon remanufacturing CLSC
Zhang et al. [24]	CLSC	Game theory	Raw materials collection by manufacturer/retailer/Internet recycling platform	Collection channel selection considering regulatory pressure and technological innovation
Wang et al. [25]	Recycling mode in China	Case studies	Sustainable “Internet+” recycling platform	Investigating 10 enterprises operating “Internet+” recycling in China
Jian et al. [26]	CLSC	Game theory	Recycling in a cooperative “Internet+ recycling” platform	Collaborative collection strategies in the “Internet+ recycling” business model
Chen et al. [29]	CLSC	Game theory and optimization	Recycling by the manufacturer or the retailer	Remanufacturing process innovation considering cost-sharing mechanism and different power structures
Xiang and Xu [23]	Two stage re-CLSC	Game theory	Recycling by the Internet recycling platforms	Dynamic game strategies considering big data, technological innovation and overconfidence
Lotfi et al. [31]	Sustainable CLSC	Robust optimization	Recycling by the manufacturer or the retailer	SC network design with Lagrange relaxation and fixand-optimize
Samuel et al. [32]	CLSC	Robust optimization	Recycling by the recycling center	Effects of the return quality on CLSC under carbon policies
This paper	CLSC	Game theory	Recycling in a cooperative “Internet+ recycling” platform or by the 3PR	Platform participation strategy of “Internet+ recycling” platform

**Table 2:** All parameters and variables used in this research.

Notation	Definition
Parameters	
*m*	Unit material and manufacturing cost
*c_s_*	Unit cost for new parts
*c_m_*	Unit manufacturing cost for new products
*λ*	Disassembly rate for used products
*d*	Unit processing cost of disassembly rate for used products
*f*	Unit buyback fee for high-quality parts
*g*	Unit buyback fee for low-quality parts
*α*	Market size
*β*	Sensitivity coefficient of sale price to demand
*a*	Sensitivity coefficient of recycling price to recycling quantity
*b*_1_	Sensitivity coefficient of recycling effort level by the 3PR to recycling quantity
*θ*_1_	Scaling parameter of recycling cost by the 3PR
*δ*	Cost-sharing proportion of recycling cost
Functions	
*D*	Demand of new products
*Q*	Recycling quantities of used products
*C_A_t__*	“Internet+ recycling” cost undertaken by the 3PR
Decision variables	
*w*	Wholesale price
*p*	Sale price
*p_r_*	Recycling price
*A_t_*	Recycling effort level by the 3PR
Extended Model	
*γ*	Sensitivity coefficient of recycling effort level by the manufacturer to demand
*b*_2_	Sensitivity coefficient of recycling effort level by the manufacturer to recycling quantity
*θ*_2_	Scaling parameter of recycling cost by the manufacturer
*C_A_m__*	“Internet+ recycling” cost undertaken by the manufacturer
*A_m_*	Recycling effort level by the manufacturer

## Data Availability

Data are contained within the article.

## Appendix A

**Proof of Lemma 1.** The Hessian matrix of $\pi_{3PR}^{N}$ over (*p_r_*, *A_t_*) is derived as follows:

$$H=\left[\begin{array}{cc} -2a & -b_{1}\\ -b_{1} & -\theta_{1} \end{array}\right],$$

From 0 < *δ* < 1, $b_{1}<\sqrt{a\theta_{1}}$, and $2a\theta_{1}-b_{1}^{2}>a\theta_{1}-b_{1}^{2}>0$, we have H_1_ = −2*a* < 0 and H_2_ = 2*aθ*_1_ − *b*_1_^2^ > 0, which is jointly concave on (*p_r_*, *A_t_*). Solving the conditions of $\partial \pi_{3PR}^{N}(p_{r},A_{t})/\partial p_{r}=0$ and $\partial \pi_{3PR}^{N}(p_{r},A_{t})/\partial A_{t}=0$ for (*p_r_*, *A_t_*), we have (*p_r_*(*w*), *A_t_*(*w*)).

(A1) $$\partial \pi_{3PR}^{N}(p_{r},A_{t})/\partial p_{r}=-A_{t}b_{1}-a[2p_{r}-(f-d-g)\lambda -g]=0$$

(A2) $$\partial \pi_{3PR}^{N}(p_{r},A_{t})/\partial A_{t}=b_{1}g(1-\lambda )+b_{1}f\lambda -[A_{t}\theta_{1}+b_{1}(p_{r}+d\lambda )]=0$$

Furthermore, inserting them into $\pi_{M}^{N}$ derives that the second-order condition of $\pi_{M}^{N}$ over p is $\partial^{2}\pi_{M}^{N}(p)/\partial p^{2}=-2\beta <0$. Then, solving the condition of $\partial \pi_{M}^{N}(p)/\partial p=0$ for *p*, we have $p(w)=[\alpha +(c_{m}+w)\beta ]/(2\beta )$. Furthermore, inserting $\left(p_{r}(w),A_{t}(w)\right)$ and p(w) into $\pi_{S}^{N}$ derives that the second-order condition of $\pi_{S}^{N}$ over *w* is $\partial^{2}\pi_{S}^{N}(w)/\partial w^{2}=-\beta <0$. Finally, solving the condition of $\partial \pi_{S}^{N}(w)/\partial w=0$ for *w* gives $w_{}^{N*}$. Furthermore, inserting $w_{}^{N*}$ into $\left(p_{r}(w),A_{t}(w)\right)$ and p(w) renders $p_{}^{N*}$, $A_{t}^{N*}$, and $p_{r}^{N*}$. Similarly, we can obtain $D_{}^{N*}$, $Q_{}^{N*}$, $\pi_{S}^{N*}$, $\pi_{M}^{N*}$, and $\pi_{3PR}^{N*}$. Furthermore, we have $\partial Q_{}^{N*}/\partial \lambda =a^{2}(f-g-d)\theta_{1}/X$ and $\partial \pi_{3PR}^{N*}/\partial \lambda =a^{2}(f-g-d)\theta_{1}M$. When *f* > *g* + *d*, we have $\partial Q_{}^{N*}/\partial \lambda >0$ and $\partial \pi_{3PR}^{N*}/\partial \lambda >0$; otherwise, $\partial Q_{}^{N*}/\partial \lambda \leq 0$ and $\partial \pi_{3PR}^{N*}/\partial \lambda \leq 0$. From 2*aθ*_1_ − *b*_1_^2^ > 0, we have $\partial D_{}^{N*}/\partial a=a\theta_{1}\lambda M(a\theta_{1}-b_{1}^{2})/X>0$ and $\partial D_{}^{N*}/\partial b_{1}=a^{2}\theta_{1}\lambda Mb_{1}^{}/(4X)>0$. □

## Appendix B

**Proof of Lemma 2.** The proof of Lemma 2 is similar to the proof of Lemma 1; we omit the proof. □

## Appendix C

**Proof of Corollary 1.** From 0 < *δ*, *λ* < 1 and Lemmas 1 and 2, we have N−M=(1−δ)[g(1−λ)+fλ]/(δX)>0; then, $w_{}^{CS*}-w_{}^{N*}=-2a^{2}\theta_{1}\lambda (N-M)/(\delta X)<0$. Similarly, we have $p_{}^{CS*}-p_{}^{N*}<0$, $A_{t}^{CS*}-A_{t}^{N*}>0$, and $p_{r}^{CS*}-p_{r}^{N*}>0$. For the demand and recycling quantities, we have $D_{}^{CS*}-D_{}^{N*}=-\beta (p_{}^{CS*}-p_{}^{N*})>0$ and $Q_{}^{CS*}-Q_{}^{N*}>0$. □

## Appendix D

**Proof of Corollary 2.** From 0 < *δ*, *λ* < 1, $b_{1}<\sqrt{a\theta_{1}}$, *d* < *f*, and Lemmas 1 and 2, we have *N* > *M*, and we have $\pi_{3PR}^{CS*}-\pi_{3PR}^{N*}=\{a^{2}d(1-\delta )\theta_{1}\lambda [2g(1-\lambda )+(2f-d-d\delta )\lambda ]\}/(2\delta X)$. Solving $\pi_{3PR}^{CS*}-\pi_{3PR}^{N*}=0$ for δ gives ${\hat{\delta }}_{1}=1$ and ${\hat{\delta }}_{2}=[2g(1-\lambda )+(2f-d)\lambda ]/(d\lambda )$. Furthermore, when $\delta <\min\{{\hat{\delta }}_{1},{\hat{\delta }}_{2}\}$, $\pi_{3PR}^{CS*}>\pi_{3PR}^{N*}$; otherwise, $\pi_{3PR}^{CS*}\leq \pi_{3PR}^{N*}$. □

## Appendix E

**Proof of Lemma 3.** The Hessian matrix of $\pi_{3PR}^{AP}$ over (*p_r_*, *A_t_*) is derived as follows:

$$H=\left[\begin{array}{cc} -2a & -b_{1}\\ -b_{1} & -\theta_{1} \end{array}\right],$$

From 0 < *δ* < 1, $b_{1}<\sqrt{a\theta_{1}}$, and $\gamma <\min\{\sqrt{2\beta \theta_{2}},(\beta \theta_{2}X^{2}+2Z^{2})/(2XZ)\}$, we have H_1_ = −2*a* < 0 and H_2_ = 2*aθ*_1_ − *b*_1_^2^ > 0, which is jointly concave on (*p_r_*, *A_t_*). Solving the conditions of $\partial \pi_{3PR}^{AP}(p_{r},A_{t})/\partial p_{r}=0$ and $\partial \pi_{3PR}^{AP}(p_{r},A_{t})/\partial A_{t}=0$ for (*p_r_*, *A_t_*), we have (*p_r_*(*w*), *A_t_*(*w*)).

(A3) $$\partial \pi_{3PR}^{AP}(p_{r},A_{t})/\partial p_{r}=-A_{t}b_{1}-a[2p_{r}-(f-d-g)\lambda -g]-A_{m}b_{2}=0$$

(A4) $$\partial \pi_{3PR}^{AP}(p_{r},A_{t})/\partial A_{t}=b_{1}g(1-\lambda )+b_{1}f\lambda -[A_{t}\theta_{1}+b_{1}(p_{r}+d\lambda )]=0$$

Furthermore, inserting them into $\pi_{M}^{AP}$ and the Hessian matrix of $\pi_{M}^{AP}$ over (*p*, *A_m_*) derives the following:

$$H=\left[\begin{array}{cc} -2\beta & \gamma \\ \gamma & -\theta_{2} \end{array}\right],$$

From 0 < *δ* < 1, $b_{1}<\sqrt{a\theta_{1}}$, and $\gamma <\sqrt{2\beta \theta_{2}}$, we have H_1_ = −2*𝛽* < 0 and H_2_ = 2*𝛽θ*_2_ − *γ*^2^ > 0, which is jointly concave on (*p*, *A_m_*). Then, solving the conditions of $\partial \pi_{M}^{AP}(p,A_{m})/\partial p=0$ and $\partial \pi_{M}^{AP}(p,A_{m})/\partial A_{m}=0$ for (*p*, *A_m_*), we have (*p*(*w*), *A_m_*(*w*)).

(A5) $$\partial \pi_{M}^{AP}(p,A_{m})/\partial p=-\alpha +\beta (2p-c_{m}-w)-\gamma A_{m}=0$$

(A6) $$\partial \pi_{M}^{AP}(p,A_{m})/\partial A_{m}=(p-c_{m}-w)\gamma -\theta_{2}A_{m}+ab_{2}(w-f)\theta_{1}\lambda /X=0$$

Furthermore, inserting (*p_r_*(*w*), *A_t_*(*w*)) and *p*(*w*) into $\pi_{S}^{AP}$, the second-order condition of $\pi_{S}^{AP}$ over *w* is derived as $\partial^{2}\pi_{S}^{AP}(w)/\partial w^{2}=-2\beta \cdot [\beta \theta_{2}X^{2}+2Z^{2}-2XZ\gamma ]/(X^{2}Y)$. From $\gamma <\min\{\sqrt{2\beta \theta_{2}},(\beta \theta_{2}X^{2}+2Z^{2})/(2XZ)\}$, we have $\partial^{2}\pi_{S}^{AP}(w)/\partial w^{2}<0$. Finally, solving the condition of $\partial \pi_{S}^{AP}(w)/\partial w=0$ for *w* gives $w_{}^{AP*}$. Furthermore, inserting $w_{}^{AP*}$ into (*p_r_*(*w*), *A_t_*(*w*)) and *p*(*w*) renders $p_{}^{AP*}$, $A_{m}^{AP*}$, $A_{t}^{AP*}$, and $p_{r}^{AP*}$. Similarly, we can obtain $D_{}^{AP*}$, $Q_{}^{AP*}$, $\pi_{S}^{AP*}$, $\pi_{M}^{AP*}$, and $\pi_{3PR}^{AP*}$ Furthermore, from $\pi_{3PR}^{AP*}=\theta_{1}{\left\{b_{2}A_{m}^{AP*}+a[g+(f-d-g)\lambda ]\right\}}^{2}/(2X)$, we have that $\pi_{3PR}^{AP*}$ is increased with $A_{m}^{AP*}$. □

## Appendix F

**Proof of Corollary 3.** From 0 < *δ*, *λ* < 1, $b_{1}<\sqrt{a\theta_{1}}$, *d* < *f*, and Lemmas 1 and 3, we have $\pi_{3PR}^{AP*}-\pi_{3PR}^{N*}=\theta_{1}b_{2}A_{m}^{AP*}/(2X)>0$. From Corollary 2, we have that when $\delta <\min\{{\hat{\delta }}_{1},{\hat{\delta }}_{2}\}$, $\pi_{3PR}^{CS*}>\pi_{3PR}^{N*}$; otherwise, $\pi_{3PR}^{CS*}\leq \pi_{3PR}^{N*}$. Therefore, there must be a threshold ${\hat{\delta }}_{3}$ that when ${\hat{\delta }}_{3}>\min\{{\hat{\delta }}_{1},{\hat{\delta }}_{2}\}$, $\pi_{3PR}^{AP*}>\pi_{3PR}^{CS*}$. □
